# T2* Placental Magnetic Resonance Imaging in Preterm Preeclampsia: An Observational Cohort Study

**DOI:** 10.1161/HYPERTENSIONAHA.120.14701

**Published:** 2020-04-27

**Authors:** Alison E.P. Ho, Jana Hutter, Laurence H. Jackson, Paul T. Seed, Laura Mccabe, Mudher Al-Adnani, Andreas Marnerides, Simi George, Lisa Story, Joseph V. Hajnal, Mary A. Rutherford, Lucy C. Chappell

**Affiliations:** 1 From the Department of Women and Children’s Health, School of Life Course Sciences (A.E.P.H., P.S., L.S., L.C.C.), King’s College London, United Kingdom; 2 Centre for the Developing Brain (J.H., L.H.J., L.M., J.V.H., M.A.R.), King’s College London, United Kingdom; 3 Biomedical Engineering Department (J.H., L.H.J., J.V.H.), King’s College London, United Kingdom; 4 Department of Cellular Pathology, Guy’s and St Thomas’ Hospital, London, United Kingdom (M.A.-A., A.M., S.G.).

**Keywords:** magnetic resonance imaging, placenta, preeclampsia, pregnancy

## Abstract

Placental dysfunction underlies the cause of pregnancies complicated by preeclampsia. The use of placental magnetic resonance imaging to provide an insight into the pathophysiology of preeclampsia and thus assess its potential use to inform prognosis and clinical management was explored. In this prospective observational cohort study, 14 women with preterm preeclampsia and 48 gestation-matched controls using 3-Tesla magnetic resonance imaging at median of 31.6 weeks (interquartile range [IQR], 28.6–34.6) and 32.2 weeks (IQR, 28.6–33.8), respectively, were imaged. The acquired data included T2-weighted images and T2* maps of the placenta, the latter an indicative measure of placental oxygenation. Placentae in women with preeclampsia demonstrated advanced lobulation, varied lobule sizes, high granularity, and substantial areas of low-signal intensity on T2-weighted imaging, with reduced entire placental mean T2* values for gestational age (2 sample *t* test, t=7.49) correlating with a reduction in maternal PlGF (placental growth factor) concentrations (Spearman rank correlation coefficient 0.76) and increased lacunarity values (t=3.26). Median mean T2* reduced from 67 ms (IQR, 54–73) at 26.0 to 29.8 weeks’ gestation to 38 ms (IQR, 28–40) at 34.0 to 37.9 weeks’ gestation in the control group. In women with preeclampsia, median T2* was 23 ms (IQR, 20–23) at 26.0 to 29.8 weeks’ gestation and remained low (22 ms [IQR, 20–26] at 34.0–37.8 weeks’ gestation). Histological features of maternal vascular malperfusion were only found in placentae from women with preeclampsia. Placental volume did not differ between the control group and women with preeclampsia. Placental magnetic resonance imaging allows both objective quantification of placental function in vivo and elucidation of the complex mechanisms underlying preeclampsia development.

## Abstract 2

Supplemental Digital Content is available in the text.

**See Editorial, pp 1412–1413**

Preeclampsia affects ≈3% of all pregnancies^1^ with associated maternal complications of renal injury, hepatic injury, stroke, hemorrhage, and eclampsia, as well as fetal complications, including growth restriction and stillbirth. Half of the women with severe preeclampsia deliver preterm and one in 20 stillbirths without congenital abnormality occur in women with preeclampsia.^2^

Placental dysfunction underlies the cause of pregnancies complicated by preeclampsia and their associated adverse pregnancy outcomes.^3,4^ In vivo assessment of placental function in clinical practice is currently limited to indirect assessments as a proxy for placental function, including measures of fetal growth and Doppler umbilical artery velocimetry.

Objective in vivo assessment of placental structure and quantification of function may help elucidate the pathophysiology in those pregnancies with established preeclampsia and provide a potential tool to inform prognosis. Quantitative noninvasive in vivo assessment of the placenta can be achieved with magnetic resonance imaging (MRI). T2* mapping probes placental oxygenation as the paramagnetic properties of deoxyhemoglobin cause magnetic field distortions and a faster T2* decay (ie, reduced transverse relaxation time) than oxyhemoglobin. Hypoxic tissues, therefore, have a reduced T2* value (blood oxygen level dependency effect). In addition to oxygenation, T2* values are also affected by lipid content, calcium content, extracellular fluid volume, and surface area, the latter contributed by cellular membranes, intracellular or extracellular macromolecules.^5^ Studies have shown a linear reduction in mean T2* values in uncomplicated pregnancies with increasing gestation^6^ and an increase in placental T2* with maternal hyperoxia.^7,8^ Lower mean T2* values have been found in placentae of pregnancies complicated by fetal growth restriction^9,10^; however, to our knowledge, no published study has reported T2* evaluation in women with preeclampsia.

The aim of this study was to explore the use of placental MRI to provide an insight into the pathophysiology of preeclampsia and thus assess its potential use to inform prognosis and clinical management.

## Methods

The data set will be made available from the corresponding author upon reasonable request, with input from the investigator group where applicable, and in accordance with the data sharing policies of King’s College London. The acquisition and processing pipeline for T2* mapping has been published and is freely available.^6^

### Study Design

This prospective observational cohort study was undertaken at St Thomas’ Hospital, London, a tertiary-level maternity unit. Women with preeclampsia were approached in person antenatally. Women in the control group were recruited at their routine 20-week anomaly scan or self-referred to take part in the study. All women in the study gave written informed consent.

Follow-up was until delivery. Prospectively specified data collection included baseline demographic characteristics, maternal, and neonatal outcomes.

Women were considered for inclusion in the study if they had a singleton pregnancy, were clinically stable, over 16 years of age, not claustrophobic with no contraindication for MRI. They were assessed for our Philips 3T Achieva scanner; given the 60 cm scanner bore size, women also had to have a body mass index <30 kg/m^2^ and abdominal girth <130 cm.

Preeclampsia was prospectively defined using the international consensus definition.^11^ This was women who developed gestational hypertension accompanied by one or more of the following new-onset conditions at or after 20 weeks’ gestation: proteinuria, acute kidney injury, liver involvement, neurological complications, hematological complications, and uteroplacental dysfunction. Preeclampsia superimposed on chronic hypertension was defined as the additional presence of maternal organ dysfunction consistent with preeclampsia. PlGF was not used as part of the clinical or research definition. Clinical management of hypertensive women was according to national guidelines,^12^ with responsibility under the attending consultant.

Women in the control group fulfilled the following criteria: no diagnosis of a hypertensive disorder at enrollment and until delivery, no significant past medical history, no pregnancy complications (including gestational diabetes mellitus), delivery at term with birthweight between the third and 97th centile (calculated using International Fetal and Newborn Growth Consortium for the 21st Century version 1.3.5)^13^ thus excluding potential confounders of placental change.^14–17^ These women were gestation matched to women with preeclampsia and selected from a research study (Placenta Imaging Project, REC 16/LO/1573, IRAS 201609), the primary objective of which is to develop a novel magnetic resonance approach to assess growth and development of the human placenta. Gestation matching was achieved masked to values derived from MRI so that 3 women with uncomplicated pregnancies with imaging within 2 weeks of the gestation at which each woman with preeclampsia was imaged were chosen for comparison.

No formal sample size was calculated for power of outcome variables as this was an exploratory study describing a novel technique in technology development application. The study was approved by the Fulham Research Ethics Committee, REC 16/LO/1573.

### Magnetic Resonance Imaging

MRI was performed on a clinical Philips 3T Achieva with a 32-channel cardiac coil. Women underwent MRI on up to 2 occasions, a minimum of 2 weeks apart and at any time point between their clinically routine anomaly ultrasound scan (at around 18–22 weeks’ gestation) and delivery. Imaging was performed supine with supported lower limbs and shoulders after a period of 3 minutes in left lateral. Total imaging time did not exceed 1 hour, and women were offered a break of under 30 minutes halfway through the scan. Maternal assessments during imaging included blood pressure measurements every 10 minutes with continuous maternal heart rate and oxygen saturation monitoring. All scans had an obstetrician or specialist midwife present throughout. No pharmacological sedation was used.

Image-based shimming was achieved using an in-house tool, after acquiring a B0 map, to reduce the effect of inhomogeneities in the magnetic field. Multi-echo gradient echo, echo planar imaging at 3 mm^3^ resolution was performed with free breathing and took <1 minute, with the whole placenta covered within 60 slices (5 echo times: 13.81 ms/70.40 ms/127.00 ms/183.60 ms/240.2 ms, repetition time=3 seconds, SENSitivity Encoding=3, halfscan=0.6). One scan was performed at 2 mm3 resolution. Echo times result from the chosen EPI train characteristics. Thereby, the intra-echo spacing was chosen to minimize acoustic noise and the interecho spacing as the minimal possible spacing. Parameters were kept constant between women with preeclampsia and the control group.

A long TE (180 ms) T2-weighted single-shot turbo spin-echo sequence of the whole uterus (thereby including placenta) was acquired in coronal and sagittal planes to the mother with repetition time=16 seconds, SENSitivity Encoding=2.5, and partial Fourier 0.625. In-plane resolution was 1.5 mm × 1.5 mm, slice thickness 2.5 mm with an overlap of 0.5 mm. The field of view was 300×360×[100−200] mm (coronal) and 300×300×340 mm (sagittal) in the FH×RL×AP directions, respectively. Structural images of the fetal brain were reported and available to the clinical team. Data quality was assessed to exclude sequences with uterine contractions. Visual analysis examined the signal intensity across the placenta and whether the presence of placental lobules and septae were visually apparent. The signal intensity within lobules was visually assessed for granularity with high granularity defined as the presence of both high- and low-signal intensity within individual lobules.

An in-house Python script by J. Hutter produced T2* maps. The placenta was manually segmented by an experienced observer (A.E.P. Ho) using ITK-SNAP. A further processing step calculated mean placental T2*and lacunarity. Lacunarity values reflect the spatial distribution of gaps of a specific size within lobules. The acquisition and processing pipeline for T2* imaging has been described previously and shown to have good reproducibility with a high Dice coefficient between observers who segmented the placenta.^6,18^

### Placental Growth Factor

Venepuncture blood sampling was performed as close to MRI as feasible, usually on the same day (in 32 out of 43 available samples). Six milliliters of blood were drawn into a bottle containing ethylenediamine tetra-acetic acid, transported to the laboratory within 1 hour and underwent centrifugation at 1400*g* (rcf) for 10 minutes at 4°C. PlGF was quantified using the Triage PlGF Test (Alere, San Diego, CA) according to the manufacturer’s instructions while masked to both cohort and clinical outcome. The clinical team did not receive the result; however, in 5 women with preeclampsia PlGF was performed as part of their clinical care as diagnostic workup^12^ when preeclampsia was suspected.

### Placental Histology

Following delivery and where available, placentas underwent histological examination according to local protocols at the Cellular Pathology Department, St Thomas’ Hospital. Placentas were fixed in 10% buffered formalin and trimmed of both umbilical cord and membranes for placenta weight. The following areas were sampled and then embedded in paraffin: 2 transverse sections of the umbilical cord, one roll of membranes (including rupture site), 2 to 3 full-thickness blocks of the placental parenchyma away from the placental edge (including fetal and maternal surfaces). Additional areas were sampled depending on macroscopic findings. Paraffin-embedded tissue sections were then cut into 4-micron sections, deparaffinized and stained with hematoxylin and eosin before histological examination. A clinical report for all placentas submitted was issued, in accordance with local hospital reporting guidelines. Histological slides were then reexamined for features of maternal vascular malperfusion, fetal vascular malperfusion, and acute chorioamnionitis by histopathologists who were masked to the pregnancy outcome; features were identified and classified using guidelines from the International Placental Pathology Consensus Meeting, Amsterdam 2014.^19^ Any discrepancies between reporting histopathologists were reexamined (again masked to the pregnancy outcome) and a consensus opinion was reached.

### Statistical Methods

In uncomplicated pregnancies, gestation-adjusted reference ranges for placental mean T2* and lacunarity values were established after examining a range of possible models using the Stata command -xriml-,^20^ and 10% to 90% reference range established. A 2 sample *t* test was used to compare placental mean T2* and lacunarity (z scores) between these groups, using robust standard errors to allow for clustering given 2 women were imaged twice in the preeclamptic group. Multiple regression, adjusting for gestation, was used to compare placental volume between uncomplicated pregnancies and those with preeclampsia.

Spearman rank correlation coefficient was calculated to evaluate the relationship between PlGF and placental mean T2*. Women who declined venepuncture were not included in the analysis. Birthweight centiles were calculated using International Fetal and Newborn Growth Consortium for the 21st Century version 1.3.5.^13^ Pregnancy outcome data were available for all women in both cohorts. Statistical analysis was performed using Stata version 15.1 (StataCorp, College Station, TX).

## Results

Enrollment was between May 2017 and April 2019. Of the 14 women with preeclampsia who were recruited, all 14 women were imaged (none of whom had uterine contractions during imaging) with 48 gestation-matched controls. Five of 14 women (36%) had superimposed preeclampsia on a background of chronic hypertension (Table 1). Women with preeclampsia had higher blood pressures both prior and during imaging and had lower PlGF concentrations (Table 1). Of women with available data on hemoglobin concentrations, the median hemoglobin at booking was 123 g/L (interquartile range, 121–131) and at 28 weeks’ gestation was 117 g/L (interquartile range, 113–122; n=37 women). In women with preeclampsia, the median hemoglobin at booking was 124 g/L (interquartile range, 120–131) and at 28 weeks’ gestation was 120 g/L (interquartile range, 110–137; n=10 women).

**Table 1.**
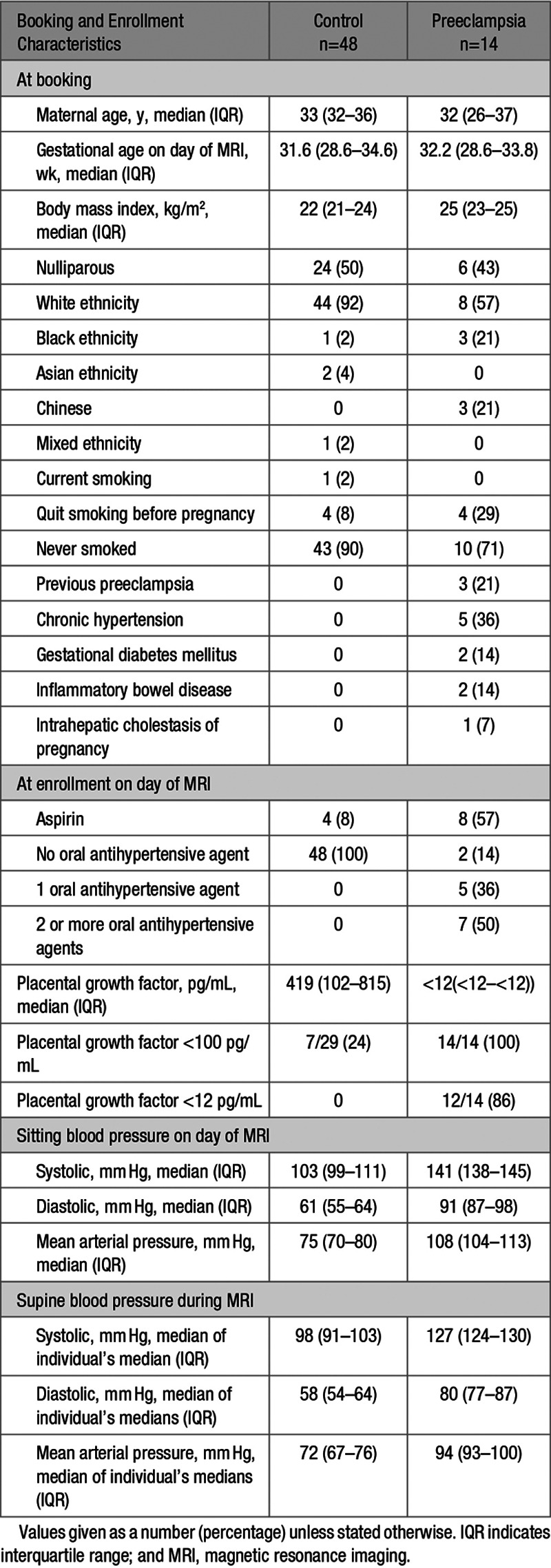
Characteristics at Booking and Enrollment

Time from MRI to delivery was shorter in women with preeclampsia (Table 2). In the preeclamptic cohort, 11 women delivered preterm with 7 before 34 weeks’ gestation (Table 2, Table S1 in the Data Supplement) while all women in the control cohort delivered after 37 weeks’ gestation. Twenty-one placentae from the control group and 12 placentae from the preeclamptic group underwent histological examination, with double reading by 2 histopathologists to standardized protocols, masked to clinical outcome (Table 3). On assessment, there were no features of maternal vascular malperfusion on placental histological examination in the control group while 10 of 12 placentae examined from women with preeclampsia did (Table 3). In the 2 placentae from preeclamptic women with no features of maternal vascular malperfusion, delivery occurred after 37 weeks’ gestation with normal birthweight centiles. There were no features of fetal vascular malperfusion on placental histopathologic examination in either cohort.

**Table 2.**
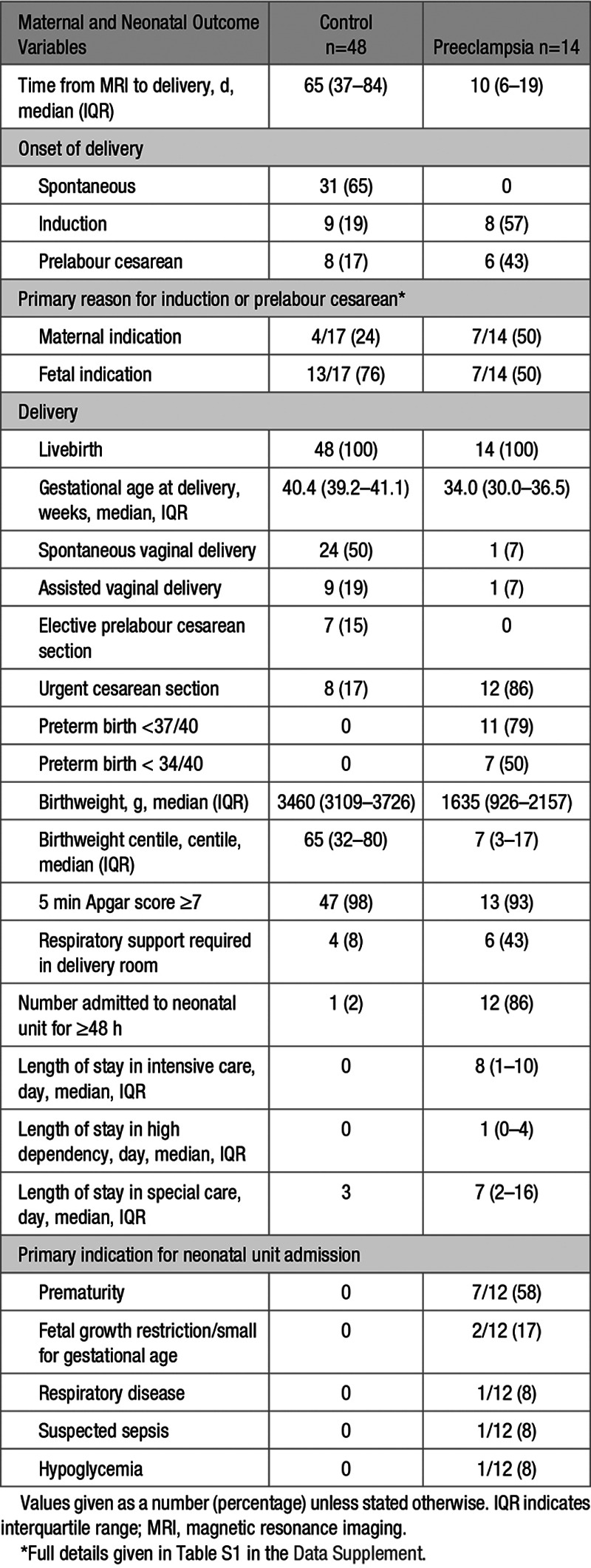
Maternal and Neonatal Outcomes

**Table 3.**
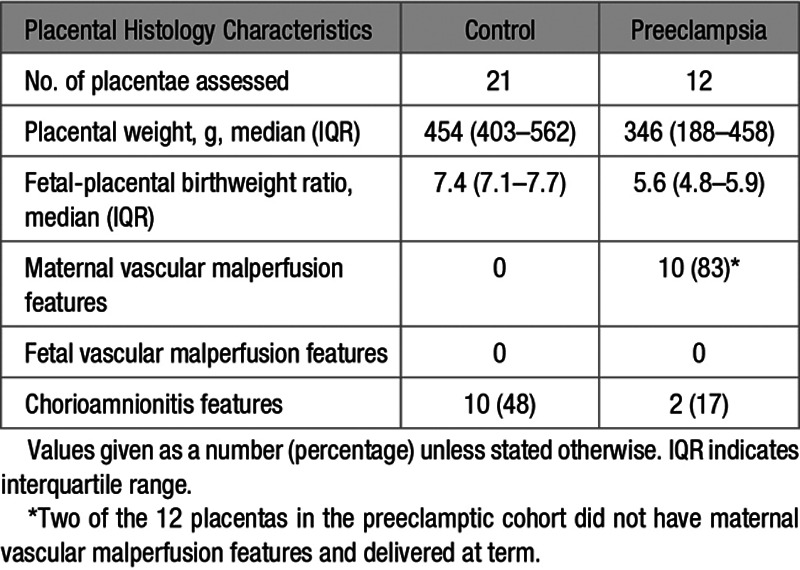
Placental Histology Findings

**Figure 1.**
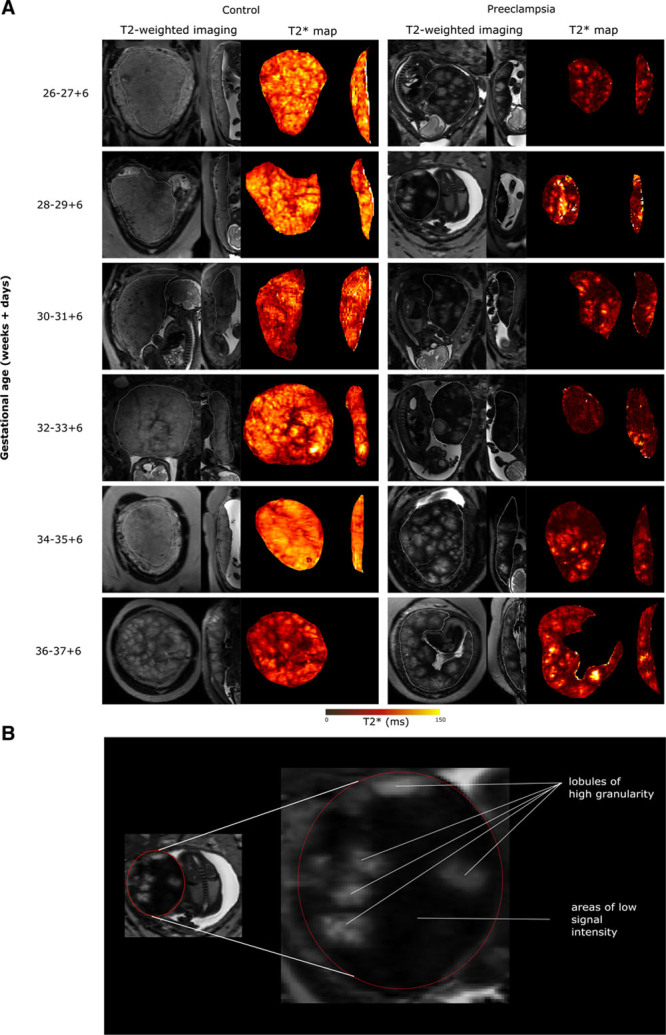
T2-weighted imaging and T2* maps. **A**, Example T2-weighted imaging and T2* maps in coronal and sagittal planes across gestation. **B**, Features of T2-weighted imaging in women with preeclampsia.

Example T2-weighted images from the control and preeclamptic cohorts are shown in Figure 1, with areas of high signal on T2-weighted imaging corresponding to long T2* and low signal on T2-weighted imaging corresponding to short T2* values. In the control cohort, placental lobularity (the visual presence of lobules, ie, presumed functional units) was more apparent with increasing gestational age at imaging. The placental lobules were of low granularity (ie, consistent signal intensity within each lobule). Compared to gestation-matched controls, the placentae in women with preeclampsia showed more marked lobularity (Figure 1, with numerical data given in Table S2 in the Data Supplement), variable lobule size and high granularity with a decline in T2* towards lobule periphery. In addition, the placentae in women with preeclampsia had substantial additional areas of low-signal intensity (Figure 1).

**Figure 2.**
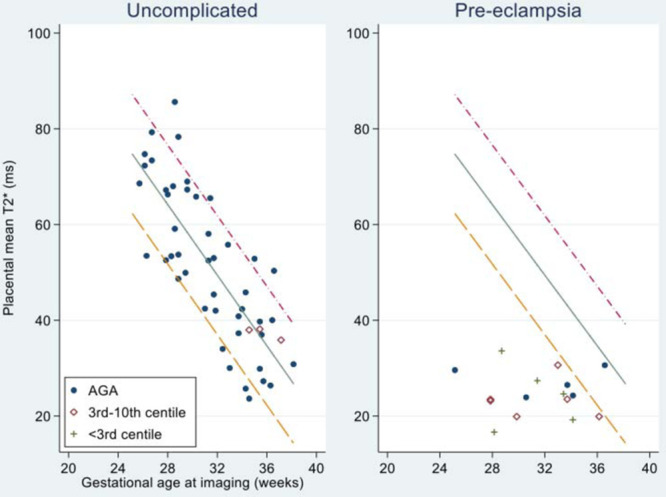
Scatterplot of placental mean T2* against gestational age at imaging, lines representing 10th, 50th, and 90th centiles. Actual placental mean T2* values given in Table S2 in the Data Supplement.

**Figure 3.**
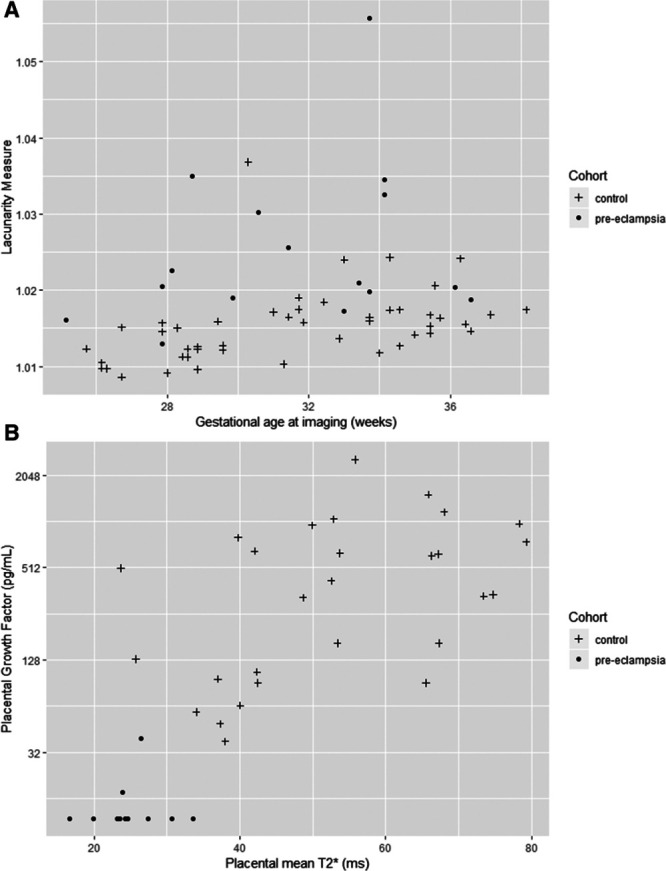
MRI derived measures and placental growth factor concentrations in both groups (control and preeclampsia). **A**, Scatterplot of lacunarity measure (derived from T2* mapping) against gestational age at imaging. Actual placental lacunarity values given in Table S2 in the Data Supplement. **B**, Scatterplot of PlGF (placental growth factor; sample taken within 2 wk of magnetic resonance imaging) against placental mean T2* (derived from T2* mapping).

With increasing gestation, placental mean T2* values decreased linearly in the control cohort (Figure 2, Table S2 in the Data Supplement). In women with preeclampsia, 13 of 14 cases had placental mean T2* values lower than the 10th centile of normal values derived from the gestation-matched control cohort (2 sample *t* test, t=7.49 versus controls). One woman with preeclampsia and placental mean T2* value within normal range delivered a baby of normal birthweight centile at 37 weeks’ gestation, with normal placental histological examination. Women with preeclampsia had higher placental lacunarity values (2 sample *t* test, t=3.83) on T2* and in the control group, lacunarity increased with increasing gestation (Figure 3A, Table S2 in the Data Supplement). Placental mean T2* positively correlated (Spearman rank correlation coefficient of 0.76) with PlGF concentration (Figure 3B). Placental volume did not differ significantly between women in the control group and women with preeclampsia (Figure S1 in the Data Supplement). Two women with preeclampsia were imaged twice, 2 weeks apart (as indicated in the participant flow diagram in Figure S2 in the Data Supplement).

## Discussion

Using a well-tolerated optimized MRI protocol,^18^ this study has provided a magnetic resonance whole placental phenotype in pregnancies complicated by preeclampsia, and these findings have been compared to a gestation-matched control cohort.

On visual inspection of T2-weighted imaging, placentae from women with preeclampsia showed substantial areas of low-signal intensity, advanced lobularity, and high granularity within lobules. Objective magnetic resonance quantification of these placentae also showed a reduced entire placental mean T2* for gestational age, with higher lacunarity values compared with controls. The placental volume in the preeclamptic group was not significantly different to those in the control group.

A major strength of this study is the novelty of the imaging undertaken in women with preeclampsia, particularly assessing placental mean T2* with a measure of texture (lacunarity values) in a cohort of women with preeclampsia. Visual analysis can be easily adopted in clinical practice, without the need for further processing of imaging data. However, while simple visual analysis of T2-weighted images alone showed characteristic features of the placenta in women with preeclampsia, T2* information obtained objective quantifiable information allowing comparisons between women, among which the whole placental mean T2* was the most discriminatory in identifying women with preeclampsia. Rapid acquisition of T2* images (in <1 minute) increases potential for clinical applicability and used a technique which has been previously demonstrated to be reproducible.^18^ Multi-Echo acquisition was chosen as it freezes motion per slice. All data required for the T2* fit per slice was acquired in <250 ms. The motion occurring between slices does not influence the fitting process.

Our robust safety approach included blood pressure monitoring with continuous maternal heart rate and oxygen saturation during imaging. A strength of this study is that we have imaged women at a range of gestations (25–37 weeks) in our preeclamptic cohort with anticipated complications, including fetal growth restriction, iatrogenic preterm delivery, neonatal unit admission, and low birthweight centiles. Limitations included being unable to image women with a body mass index greater than 30 kg/m^2^ due to size limitations of our 3T Achieva scanner bore; however, we have been able to image across the third trimester, thus increasing applicability to a clinical context.

In uncomplicated pregnancies, T2-weighted visual analysis shows increasing lobulation with widening of the low-signal intensity septal areas around lobules. This and previous studies have shown that this visual maturation corresponds to a reduction in mean T2* values across the placenta.^6,10,21,22^ Mean T2* values are consistent with previously reported studies^18^; however, values are inherently affected by magnetic field strength, and there remains a lack of literature reporting mean T2* in uncomplicated pregnancies at 3 Tesla.^23^ The placental volume is derived from a conservative manual segmentation of the T2* maps to ensure only placental tissue sampled, and thus is smaller than previously reported in the literature where volume is derived from T2-weighted imaging.^24,25^

Our findings of low placental mean T2* values in pregnancies complicated by preeclampsia are consistent with a previous smaller study^10^ in which T2* values were examined in 4 women with fetal growth restriction, one of whom had co-existing preeclampsia. Another study also examined T2* in a mixed cohort of 33 women (reported as R2*=1/T2*) at earlier gestations (before 24 weeks’ gestation), including 2 women with fetal growth restriction and one with preeclampsia.^21^ Mean R2* values in women with fetal growth restriction were similar to uncomplicated pregnancies. However, these women were imaged during early second trimester and all delivered at term thus perhaps suggesting a different disease mechanism in preterm preeclampsia and term preeclampsia. In our study, albeit a small sample size, co-existing fetal growth restriction in women with preeclampsia did not appear to further impact placental mean T2* values. Recent in vivo MRI in women with preeclampsia using a different imaging parameter of placental perfusion fraction (rather than T2* mapping) showed a difference between early- and late-onset preeclampsia and a decreased fraction with increasing gestation in controls correlating to maternal biomarkers.^26,27^

Contrast-enhanced imaging in Rhesus macaques has previously demonstrated a slow spread of signal intensity enhancement from a spiral artery across lobule to periphery in a naturally occurring fetal growth restriction case.^28^ No contrast was used in our study; however, we demonstrated a fall in signal intensity from the center to the periphery of lobules in women with preeclampsia. Previous studies have not included maternal biomarker analysis, while we have shown that all women with a PlGF <12 pg/mL had a mean T2* lower than gestation-matched controls.

The striking visual differences between women with preeclampsia and our control cohort on T2-weighted imaging and quantitative T2* values provide a magnetic resonance placental phenotype in preeclampsia. Marked widening of low-signal septal areas and accelerated maturation of lobules in the placentae of women with preeclampsia suggests areas of nonfunctioning or poorly functioning tissue. This is supported by detailed histological analysis which showed that 10 of 12 placentae from women with preeclampsia had evidence of maternal vascular malperfusion. Areas of low-signal intensity on T2-weighted imaging, therefore, likely reflect a poorly perfused and poorly oxygenated placenta with a corresponding short T2*, also reflected in low maternal PlGF concentrations. In a healthy placenta, these low-signal intensity areas are consistently between lobules and likely to represent normal septae with low oxygenation. In abnormal placentae, the areas of low-signal intensity on T2 are different in distribution, size and shape, suggesting poorly oxygenated areas extending beyond the normal septae. More detailed histology to selectively sample these areas would further elucidate any structural differences. All women had hemoglobin concentrations within normal limits and thus this is unlikely to have substantially affected T2* values. Within lobules, there are regional T2* differences with a marked decline in T2* towards the lobule periphery in preeclampsia. This could be explained by a reduction in perfusion of the lobule periphery with oxygenated blood following ischemic reperfusion injury from high-pressure flow in poorly remodeled spiral arteries. The peripheral areas of lobules may be more vulnerable to damage, giving rise to the areas of short T2* distal from central arteries. High lacunarity values in women with preeclampsia further support the visual phenotype seen on both T2-weighted imaging and T2* maps where there is high granularity within individual lobules and heterogeneity across the placenta.

More detailed placental histology, biomarkers relating to placental oxygenation and diffusion MRI may yield further insight into the pathophysiology of preeclampsia in conjunction with placental T2* mapping. While the relationship between concentration of deoxygenated hemoglobin and T2* values is well established (BOLD effect), it does not allow absolute quantification of oxygen concentration. T2* depends additionally on geometry, saturation, hemoglobin concentration.

A larger sample size of women with preeclampsia encompassing those with and without co-existing fetal growth restriction and those with early and late onset would be beneficial to examine potential differing mechanisms that result in additional fetal morbidity. Placental MRI may demonstrate different phenotypes in these subgroups, aligned with different placental histological findings, and require further elucidation. Further characteristics of interest would include the presence of other comorbidities, use of antihypertensive medication and aspirin usage. It would be advantageous to image using a wide bore scanner to encompass those women in whom a high body mass index and girth is more prevalent. Further studies could be useful to investigate the potential role of PlGF (and other maternal serum biomarkers of placental function, alongside conventional ultrasound parameters) for triage of clinically higher risk women in whom placental MRI may be beneficial. However, the 2 modalities (biomarkers and MRI) may be providing complementary predictive and mechanistic information.

## Perspectives

Our study using T2-weighted imaging, T2* mapping and maternal PlGF concentrations has quantitatively demonstrated a placental phenotype in preeclampsia that has helped to support prevailing theories of complex mechanisms underlying placental dysfunction in preeclampsia. Further studies to determine whether there is a potential role for predicting the subsequent development of preterm preeclampsia in high-risk groups (eg, those with chronic hypertension, gestational hypertension) or women undergoing a fetal MRI for other indications (such as fetal congenital cardiac abnormalities or fetal growth restriction) would be of interest. Future applications of T2* mapping may include monitoring of high-risk pregnancies to aid clinical management decisions or measuring effectiveness of new therapies in preeclampsia that aim to target underlying oxidative mechanisms.

## Acknowledgments

We thank all the women who participated in the study, their midwives and obstetricians involved in study recruitment. We thank Alexia Egloff for clinical reporting and the research radiographers.

## Sources of Funding

This work is funded by the National Institutes of Health (NIH) Human Placenta Project grant 1U01HD087202-01, the National Institute for Health Research (NIHR) Research Professorship (L.C. Chappell; RP-2014-05-019), Tommy’s (Registered charity no. 1060508) and Holbeck Charitable Trust with support from the Wellcome EPSRC Centre for Medical Engineering at King’s College London (WT 203148/Z/16/Z) and by the National Institute for Health Research Biomedical Research Centre based at Guy’s and St Thomas’ NHS Foundation Trust and King’s College London. P. Seed is partly funded by Tommy’s and by CLAHRC South London (NIHR). J. Hutter is funded by the Wellcome Trust through a Sir Henry Wellcome Fellowship (201374).

## Disclosures

The views expressed are those of the authors and not necessarily those of the UK National Health Service, the National Institute for Health Research, or the Department of Health and Social Care.

## Supplementary Material



## Novelty and Significance

### What Is New?

- Using in vivo magnetic resonance imaging to quantify placental function, we have evaluated and demonstrated a placental phenotype in women with pregnancies complicated by preeclampsia.
- Techniques include T2* mapping as an indicative measure of placental oxygenation and the use of lacunarity to quantify the spatial distribution of gaps of a given size across a region of interest.
- The placentae of women with preeclampsia have low mean T2* and high lacunarity values.

### What Is Relevant?

- Preeclampsia is a hypertensive disorder of pregnancy-associated with adverse pregnancy outcome.
- Placental dysfunction underlies the cause of these pregnancies.
- Magnetic resonance imaging provides objective quantification of placental function in vivo and elucidation of complex mechanisms underlying preeclampsia development, thus has potential to inform prognosis and clinical management.

### Summary

This prospective observational cohort study has used T2-weighted imaging, T2* mapping and maternal PlGF (placental growth factor) concentrations to quantitatively demonstrate a placental phenotype in preeclampsia.
