# Supplementary figures and images for: T2* Placental Magnetic Resonance Imaging in Preterm Preeclampsia: An Observational Cohort Study
Source: Hypertension. 2020 Apr 27;75(6):1523–31. doi: 10.1161/HYPERTENSIONAHA.120.14701 (PMC7682790; doi:10.1161/HYPERTENSIONAHA.120.14701)

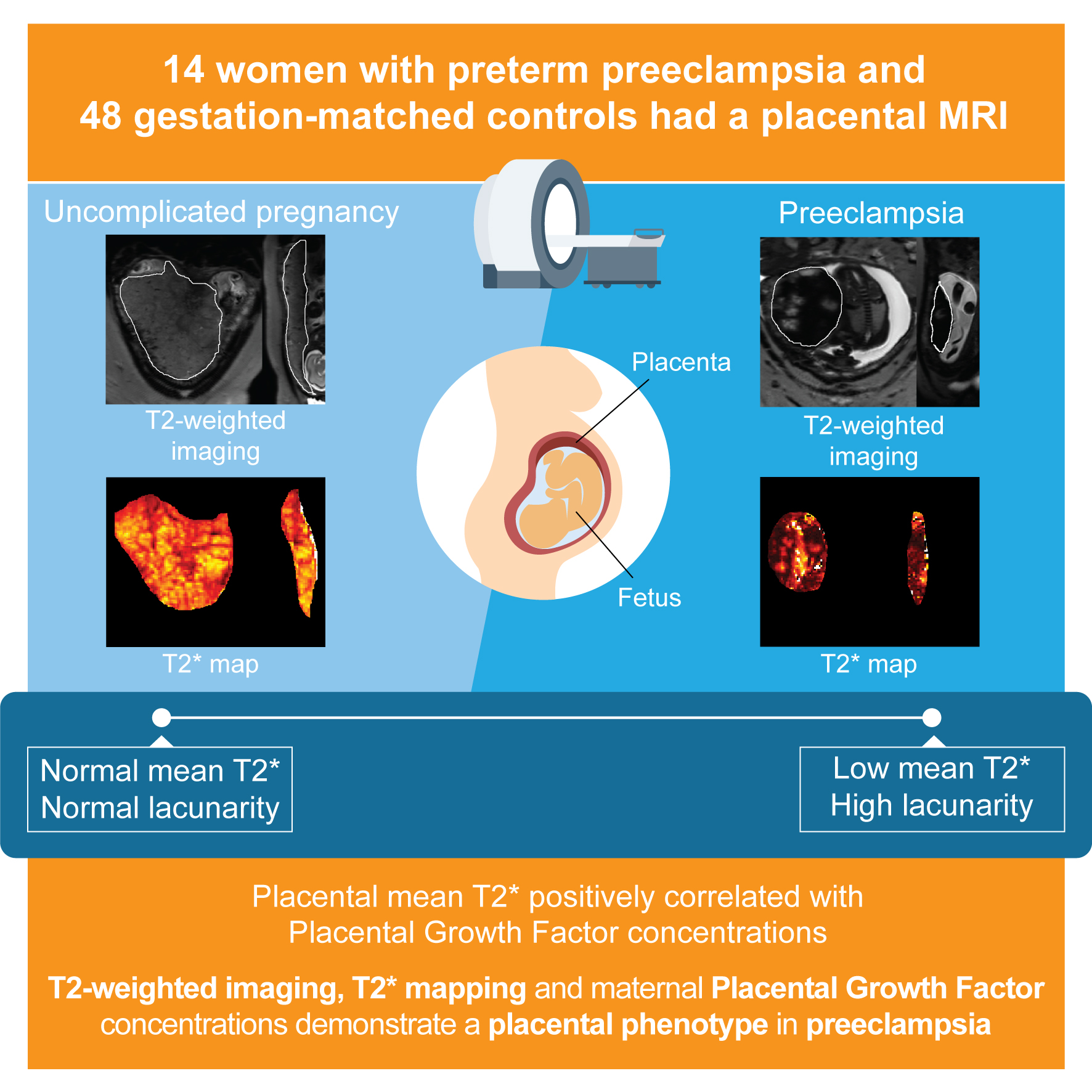

Supplement: Supplementary file 1 [file hyp-75-1523-s001.jpg]

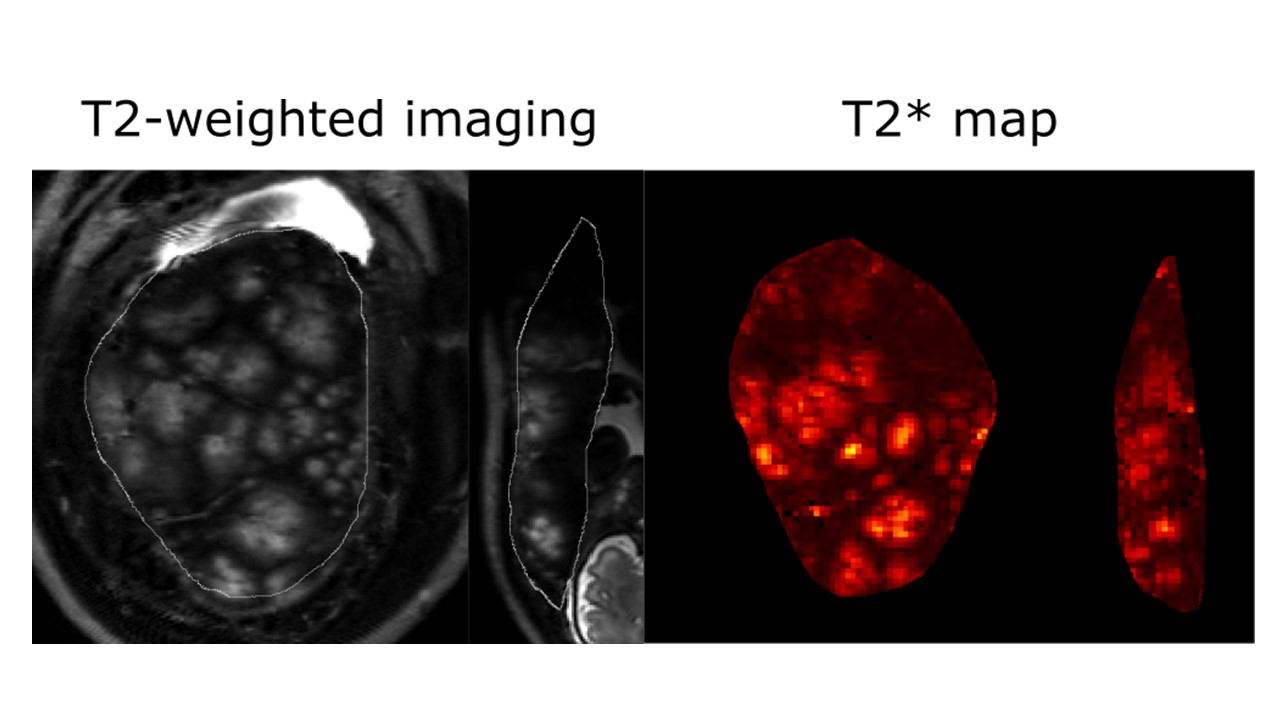

Supplement: Supplementary file 3 [file hyp-75-1523-s003.jpg]
