# Supplementary material for: T2* Placental Magnetic Resonance Imaging in Preterm Preeclampsia: An Observational Cohort Study
Source: Hypertension. 2020 Apr 27;75(6):1523–31. doi: 10.1161/HYPERTENSIONAHA.120.14701 (PMC7682790; doi:10.1161/HYPERTENSIONAHA.120.14701)
Supplement: Supplementary file 2 [file hyp-75-1523-s002.pdf]

Supplemental Data

T2* Placental Magnetic Resonance Imaging in Preterm Preeclampsia: An Observational Cohort Study

Alison E. P. Ho[1], MBBS; Jana Hutter[2,3], PhD; Laurence H. Jackson[2,3], PhD; Paul Seed[1], CStat; Laura Mccabe[2], BSc; Mudher Al-Adnani[4], MBChB; Andreas Marnerides[4], PhD; Simi George[4], MBChB; Lisa Story[1], PhD; Joseph V. Hajnal[2,3], PhD; Mary A. Rutherford[2]†, MD; Lucy C. Chappell[1]†, PhD

1. Department of Women and Children's Health, School of Life Course Sciences, King's College London, London, United Kingdom.
2. Centre for the Developing Brain, King's College London, London, United Kingdom.
3. Biomedical Engineering Department, King's College London, London, United Kingdom.
4. Department of Cellular Pathology, Guy's and St Thomas' Hospital, London, United Kingdom.

† joint senior authors

Corresponding author

Alison Ho

Email: alison.ho@kcl.ac.uk

Postal address: Department of Women and Children's Health, School of Life Course Sciences, King's College London, 10th Floor, North Wing, St Thomas' Hospital, London SE1 7EH. United Kingdom.

Supplemental Tables

Supplemental Table S1: Primary reason for induction or prelabour caesarean (taken from clinical notes).

**Primary reason for induction or prelabour caesarean**	**Control n = 17**	**Preeclampsia n=14**
Fetal growth restriction	0	5 (36)
Preeclampsia and reached 37/40	0	3 (21)
Difficult blood pressure control	0	3 (21)
Abnormal cardiotocogram	1 (6)	2 (14)
Randomised to planned delivery within trial	0	1 (7)
Prelabour rupture of membranes	5 (29)	0
Post dates	4 (24)	0
Previous caesarean/uterine surgery	2 (12)	0
Fetal presentation not cephalic	2 (12)	0
Maternal request	2 (12)	0
Placenta praevia	1 (6)	0

Values given as a number (percentage) unless stated otherwise

Supplemental Table S2: Actual placental mean T2*, lacunarity and volume values.

Placental imaging variables	26-29+6 weeks	30-33+6 weeks	34-37+6 weeks
**Preeclampsia**			
Mean T2*, ms, median (IQR)	23 (20-23)	26 (24-27)	22 (20-26)
Lacunarity, median (IQR)	1.021 (1.019-1.023)	1.023 (1.020-1.029)	1.026 (1.020-1.033)
Placental volume, ml, median (IQR)	499 (342-503)	450 (285-600)	272 (197-428)
**Control**			
Placental volume, ml, median (IQR)	427 (201-497)	321 (234-438)	385 (263-510)
Mean T2*, ms, median (IQR)	67 (54-73)	45 (41-56)	38 (28-40)
Lacunarity, median (IQR)	1.012 (1.010-1.014)	1.017 (1.016-1.019)	1.017 (1.015-1.017)

Supplemental Figures

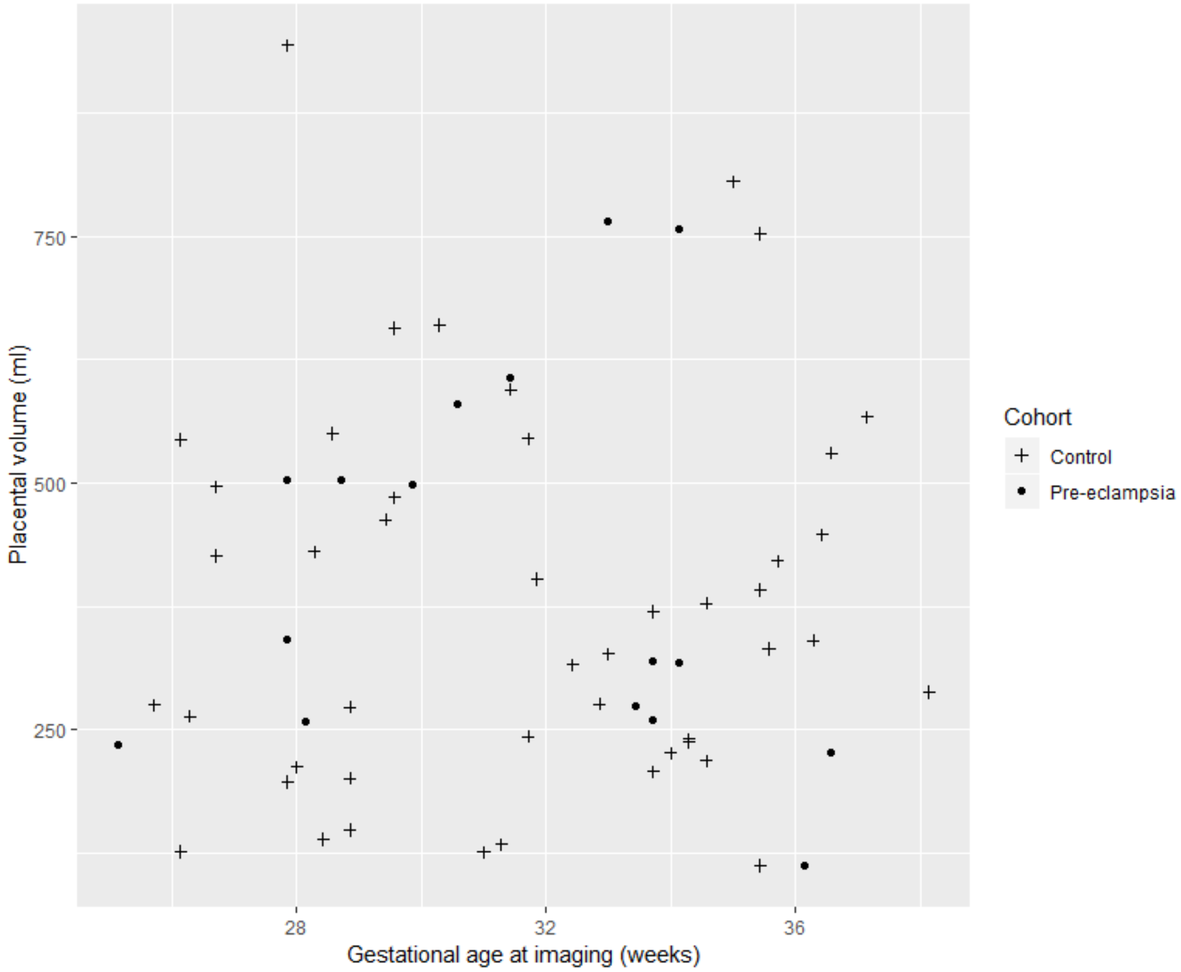

Supplemental Figure S1: Scatterplot of placental volume against gestational age at imaging.

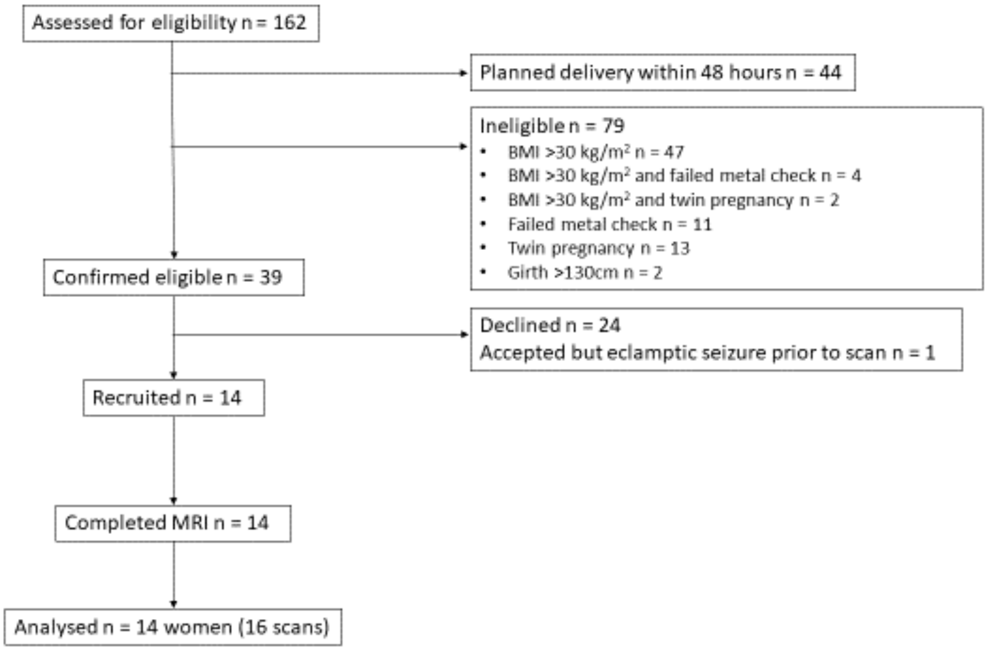

Supplemental Figure S2: Flow diagram of participants with preeclampsia.
